# The intranasal adjuvant Endocine™ enhances both systemic and mucosal immune responses in aged mice immunized with influenza antigen

**DOI:** 10.1186/s12985-017-0698-4

**Published:** 2017-03-03

**Authors:** Tina Falkeborn, Jorma Hinkula, Marie Olliver, Alf Lindberg, Anna-Karin Maltais

**Affiliations:** 1 Department of Clinical and Experimental Medicine, Linköping University, Linköping, Sweden; 2 Eurocine Vaccines AB, Karolinska Institutet Science Park, Solna, Sweden

**Keywords:** Endocine, Intranasal vaccination, Aged mice, Influenza, Mucosal adjuvant

## Abstract

Despite availability of annual influenza vaccines, influenza causes significant morbidity and mortality in the elderly. This is at least in part a result of immunosenescence; the age-dependent decrease in immunological competence that results in greater susceptibility to infections and reduced responses to vaccination. To improve protective immune responses in this age group, new vaccines strategies, such as the use of adjuvants, are needed. Here, we evaluated the mucosal vaccine adjuvant Endocine™, formulated with split influenza antigen and administered intranasally in aged (20-month old) mice. Humoral immune responses were assessed and compared to unadjuvanted intranasal and subcutaneous vaccines. We show that formulation with Endocine™ significantly enhances hemagglutination inhibition (HI) titers, as well as serum IgG and mucosal IgA antibody titers, compared to both types of unadjuvanted vaccines. Thus, our results indicate that intranasal vaccination with Endocine™ is a possible approach for the development of mucosal influenza vaccines for the elderly.

## Introduction

Worldwide, annual influenza epidemics are estimated to result in 250 000 to 500 000 deaths and up to 5 million cases of severe illness [1]. Adults 65 years of age and older are particularly vulnerable to complications from influenza infection, and account for 90% of all influenza-associated deaths in the U.S. [2]. Vaccination is the primary strategy to prevent and reduce morbidity and mortality associated with influenza [3], and the World Health Organization (WHO) recommends annual vaccination for groups at high risk of complications, including the elderly, young children, pregnant women, health-care workers, and individuals with underlying medical conditions [1]. However, it has proven difficult to stimulate potent immune responses in elderly individuals, and studies in mice, ferrets and humans show that antigen-specific immune responses decline with age [4–9]. Accordingly, a quantitative review by Goodwin et al. shows that the clinical vaccine efficacy of injectable influenza vaccines was 17–53% in the elderly [10] compared to 70–90% in younger adults [11], and that following influenza vaccination, younger adults had up to 4 times higher antibody levels compared with older adults [10].

Strategies to augment the immune response in older adults include using alternative routes of vaccine delivery, a higher dose of antigen, or addition of an adjuvant. The Fluzone^®^ High-Dose influenza vaccine (Sanofi Pasteur Inc.) was designed for people 65 years and older, and contains four times the amount of hemagglutinin (HA) contained in standard-dose vaccines [12, 13]. The adjuvanted injectable influenza vaccine Fluad^®^ was developed (by Novartis) for older adults. Both Fluzone^®^ High-Dose and Fluad^®^ have demonstrated enhanced humoral immune responses compared to standard-dose non-adjuvanted vaccines [12–15]. Nevertheless, immunization advisory committees such as the Advisory Committee on Immunization Practices (ACIP) in the U.S., and the National Advisory Committee on Immunization (NACI) in Canada, consider that there is currently insufficient evidence to make a recommendation for their routine use in the elderly population. There is currently only one intranasal influenza vaccine on the western market, which is a live attenuated influenza vaccine (LAIV) called Fluenz^®^/Flumist^®^ (MedImmune, AstraZeneca). However, LAIV is not licensed for adults >18 years in Europe, and not recommended for individuals ≥50 years in the U.S. It has been speculated that LAIV’s limited efficacy in adults and elderly could be due to its inability to infect individuals with pre-existing immunity [16–18]. Following vaccination with LAIV, viral shedding can occur and there is a possibility that the vaccine strain is transmitted [19], which could be problematic if LAIV recipients come into contact with severely immunocompromised persons.

Here we sought to evaluate a vaccine based on split influenza antigen together with the lipid-based adjuvant Endocine™ in aged mice. Endocine™ is a mucosal adjuvant that has been shown to be safe and well tolerated in both pre-clinical and clinical studies [20–24]. We have previously demonstrated that vaccines formulated with Endocine™ enhance both humoral and cell-mediated immune responses in mice after intranasal vaccination [20], and a study in ferrets demonstrated that Endocine™ induces high HI and virus neutralization titers, and fully protects ferrets from virus replication in the lungs [22]. Furthermore, a recent study by Falkeborn et al. showed that an Endocine™-adjuvanted influenza vaccine evoked serum IgG and virus neutralization titers to comparable levels as cholera toxin (CT) in mice, and induced significantly higher serum and mucosal influenza-specific IgA titers than an alum-adjuvanted vaccine administered parenterally [25]. In the current study we tested if Endocine™ could enhance systemic and mucosal humoral immune responses to influenza antigen in aged mice.

## Materials and methods

### Mice

Female BALB/c mice were purchased from Charles River, Germany and used for vaccinations at 2 or 20 months of age. All animal experiments were approved by the regional animal experimental ethics committee in Stockholm (North), Sweden. The study was performed in accordance with institutional guidelines at Adlego Biomedical AB, Stockholm, Sweden.

### Antigen and adjuvant

All mice were vaccinated with A/California/07/2009(H1N1)pdm split influenza antigen from season 2012/2013 (kindly provided by Mitsubishi Tanabe Pharma Corporation, Kanagawa, Japan) with or without the adjuvant Endocine™ (Eurocine Vaccines AB, Stockholm, Sweden), except the control groups which received saline. The adjuvant Endocine™ consists of the lipids mono-olein and oleic acid [21, 22, 24].

### Vaccination and sampling

Mice (*n* = 8-11 per group) received 3 μg HA +/- 2% Endocine™ intranasally in 5 μL/nostril, or 50 μL of 3 μg HA subcutaneously in one hind leg. During vaccination, the mice were anaesthetized with isoflurane (IsoFlo® vet, Orion Pharma Animal Health, Sollentuna, Sweden). The mice were immunized three times at three-week intervals (day 0, 21 and 42). Blood samples and nasal lavages were collected one day before each immunization and three weeks after the last immunization at termination. The samples were stored at -20 °C until analysis. After sacrifice, the lungs were removed, put in PBS and frozen at -70 °C.

### Determination of influenza-specific antibodies by ELISA

All serum samples were analyzed individually for influenza specific IgG, IgA, and subclass IgG (IgG1, IgG2a) with an enzyme-linked immunosorbent assay (ELISA). The samples were tested against the trivalent split vaccine Inflexal from season 2012/2013 (Cruzell, Madrid, Spain), consisting of A/California/07/2009 (H1N1), A/Victoria/361/2001 (H3N2) and B/Wisconsin/1/2010 influenza strains. Serological responses were measured as previously described [20] with the exception that the plates were coated with Inflexal at a concentration of 1.5 μg HA/mL. Nasal lavage was also analyzed for nasal IgA against Inflexal. These samples were incubated overnight in +4 °C on the plate and then analyzed as previously described [20]. The lungs were homogenized, flushed with PBS and the solution was collected and centrifuged to remove tissue and cell debris. To analyze total IgA, plates were coated with 1 μg/mL of Goat-anti mouse IgA (MyBiosource.com). The samples were then incubated overnight in +4 °C on the plate. To detect total IgA, Mouse Immunoglobulins AP (DAKO) diluted 1:3,000 and p-nitrophenyl phosphate (pNPP) (Sigma-Aldrich) were used. To analyze influenza specific IgA and IgG in lung homogenate, plates were coated with Inflexal and analyzed as previously described [20].

### Hemagglutination inhibition (HI)

Samples were pooled in each group from each time point except serum samples from day 63 which were analysed individually. Sera were tested in hemagglutination inhibition (HI) as previously described [26] against pH1N1 A/California/07/09 virus at Viroclinics Biosciences B.V.

### Statistical analysis

Statistical analysis was performed using GraphPad Prism (La Jolla, CA, US). Analysis of immunological parameters was performed using Kruskal-Wallis one-way ANOVA. When significant, Mann-Whitney U-test was applied for comparison between two groups. A *p*-value of <0.05 was considered statistically significant.

## Results and Discussion

### Intranasal delivery of an Endocine™-formulated influenza vaccine enhances HI and IgG titers in aged mice

**Fig. 1:**
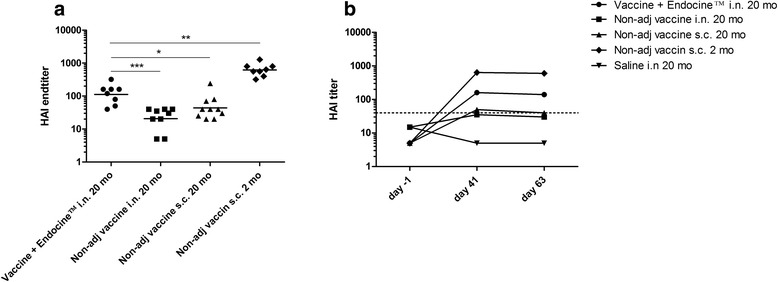
Evaluation of HI response. Mice were vaccinated intranasally or subcutaneously three times with three week intervals using split influenza antigen (A/California/07/2009(H1N1)pdm) formulated with or without the adjuvant Endocine™. **a** Individual HI titers (GMT) at day 63 and (**b**) HI titers (median) over time. Significant differences to the adjuvanted vaccine group is indicated by *p < 0.05, **p < 0.01, ***p < 0.001

Several studies have shown that by using adjuvanted vaccines in old mice, it is possible to stimulate a higher and more protective antibody response compared to non-adjuvanted vaccines [27–30]. Previous publications regarding immunization with the adjuvant Endocine™ in 2 month old mice have shown significantly increased humoral and cell-mediated immune response compared to non-adjuvanted influenza vaccine given intranasally [20, 21, 25]. To assess the humoral immune response in aged mice, we immunized 20-month old mice intranasally with antigen from the A/California/07/2009(H1N1)pdm strain formulated with or without Endocine™. In addition, both aged and young (2-month old) mice were subcutaneously immunized with unadjuvanted influenza antigen. As expected, HI titers were considerably greater in young mice compared to old mice among the subcutaneously immunized mice (Fig. 1a and b). Interestingly, addition of Endocine™ to the intranasally-administered vaccine significantly enhanced HI titers in aged mice (Fig. 1a and b). Furthermore, among the aged mice, we found that those vaccinated intranasally with the Endocine™-adjuvanted vaccine had significantly higher HI titers than mice vaccinated subcutaneously without adjuvant (Fig. 1a and b).

**Fig. 2:**
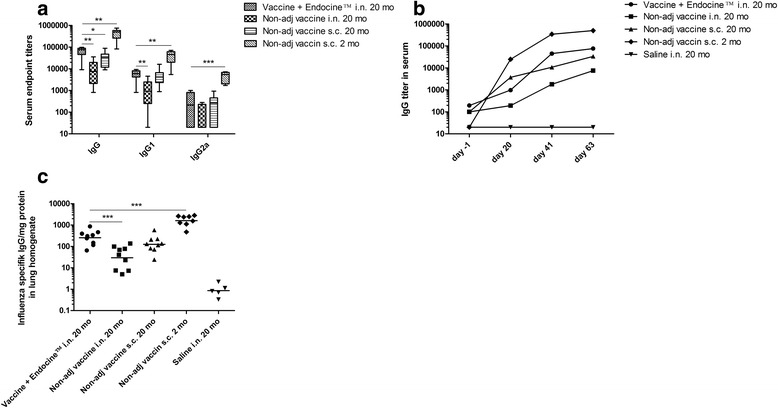
Evaluation of influenza-specific IgG responses. Mice were vaccinated intranasally or subcutaneously three times with three week intervals using split influenza antigen (A/California/07/2009(H1N1)pdm) formulated with or without the adjuvant Endocine™. Influenza-specific (**a**) serum IgG, IgG1 and IgG2a endpoint titers (GMT ± CI95), (**b**) serum IgG titer (median) over time and (**c**) IgG titers (GMT) in lung homogenates are shown. Significant differences to the adjuvanted vaccine group is indicated by *p < 0.05, **p < 0.01, ***p < 0.001

When serum IgG titers were evaluated in aged mice, we found that the Endocine™-vaccinated mice responded with significantly higher IgG and IgG1 titers than mice vaccinated intranasally without adjuvant (Fig. 2a and b), in accordance to what has previously been demonstrated in young mice [20, 21, 25]. Subcutaneously vaccinated aged mice had higher serum IgG levels than Endocine™-vaccinated mice after the first dose. Nevertheless, we noted that the Endocine™-vaccinated aged mice exhibited higher IgG titers after the second and third dose (Fig. 2a and b), indicating that two intranasal vaccine doses are needed to achieve full effect. We speculate that two vaccine doses in influenza-naïve individuals may correspond to one vaccine dose in the adult population which have pre-existing immunity against influenza viruses. In earlier studies [20] and unpublished data using 2 month old BALB/c mice, the IgG2a titers were lower than IgG1 titers. Therefore, it was expected that the IgG2a levels would be low in this study, especially since aged mice have an impaired activation of immune cells connected to the adaptive immune response [31, 32]. In a study by Higgins et al. [27], lower levels of IgG2a were also detected in aged mice compared to young mice after influenza vaccination with adjuvant. Furthermore, in a study of healthy adults aged <40 (young), 40–64 (middle-aged) and ≥65 (elderly) years, it was reported that aging was associated with a significant impairment of IgG1 antibody production (corresponds to IgG2a in mice) [33].

Analysis of influenza-specific IgG in lung homogenates of aged mice revealed that the Endocine™-adjuvanted vaccine generated significantly higher antibody titers than the unadjuvanted intranasal vaccine (Fig. 2c). Overall, our results demonstrate that intranasal delivery of an Endocine™-formulated influenza vaccine can improve vaccine immunogenicity and help overcome the limitations of immunosenescence.

### Endocine™ boosts influenza-specific mucosal IgA titers in aged mice

**Fig. 3:**
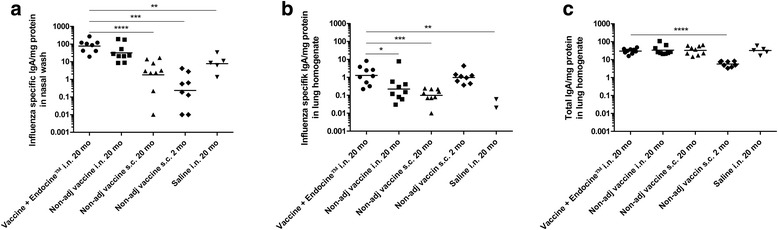
Evaluation of influenza-specific IgA response. Mice were vaccinated intranasally or subcutaneously three times with three week-interval using split influenza antigen (A/California/07/2009(H1N1)pdm) with or without the adjuvant Endocine™. Influenza-specific IgA (**a**) in nasal wash and (**b**) lung homogenate. **c** Total IgA in lung homogenate. Data shown represent geometric mean titers (GMT). For data which included values of 0 (1 mouse in the non-adj s.c. 20 month group, and 2 mice in the non-adj s.c. 2 month group), a value of 0.01 was added to calculate GMT. Significant differences to the adjuvanted vaccine group is indicated by *p < 0.05, **p < 0.01, ***p < 0.001

In addition to a systemic immune response, influenza vaccines should optimally induce a mucosal immune response in the respiratory tract. We found no influenza-specific nasal IgA in subcutaneously vaccinated mice, neither in young nor aged mice. By contrast, intranasal vaccination of aged mice potently induced influenza-specific nasal IgA (Fig. 3a). Although not statistically significant, and not to the same extent as previously seen in young mice [20, 25] and unpublished data, formulation with Endocine™ somewhat enhanced the IgA titers (Fig. 3a). Endocine™-vaccinated mice responded with 2.4 times higher geometric mean titer than mice immunized intranasally without adjuvant (GMT of 77 and 32, respectively). Interestingly, a study by Asanuma et al. showed that influenza virus colonization was totally prevented in the presence of virus-specific S-IgA antibody response in aged mice, despite their reduced levels of IgG [34].

Influenza-specific IgA levels were substantially lower in lung homogenates than in nasal washes, nevertheless, Endocine™-vaccinated mice had significantly higher IgA titers compared to mice immunized intranasally or subcutaneously without adjuvant (Fig. 3b). The total amount of IgA antibodies, not only influenza-specific, was similar in all groups of aged mice, whereas significantly lower levels were detected in the group with young mice (Fig. 3c). This is not completely unexpected, as previous analyses have shown a similar difference in IgA levels between young and older mice in gut tissue [35, 36]. Interestingly, even though the frequency of local mucosal B cells in gut tissue is reduced in elderly mice, the synthesis and secretion of immunoglobulins was shown to be higher in older mice compared to young mice [35]. Taken together, these data indicate that intranasal administration of influenza vaccine adjuvanted with Endocine™ may have advantages over parenteral immunization due to its ability to induce mucosal IgA.

In this study, challenge was not performed, however, a previous study in young mice vaccinated intranasally with influenza and Endocine™ did show reduced levels of viral RNA in the lungs [21]. Furthermore, immunization of 12 month old ferrets with influenza vaccine adjuvanted with Endocine™ induced a broad antibody response and fully protected the animals after challenge with influenza virus [22]. Future studies should explore whether intranasal immunization with Endocine™ confers protection against influenza challenge in aged mice.

The current study is one of few to evaluate the humoral systemic and mucosal immune responses to influenza vaccination in 20-month old mice. We show that it is possible to improve the immune response to an inactivated intranasal vaccine by formulating the vaccine with Endocine™. In addition, the intranasal Endocine™-adjuvanted influenza vaccine improved immune responses (HI titers, serum IgG and mucosal IgA) in aged mice compared to a non-adjuvanted parenteral vaccine.
